# Expression of Pluripotency Factors OCT4 and LIN28 Correlates with Survival Outcome in Lung Adenocarcinoma

**DOI:** 10.3390/medicina60060870

**Published:** 2024-05-26

**Authors:** Pinelopi Bosgana, Sophia Nikou, Foteinos-Ioannis Dimitrakopoulos, Vasiliki Bravou, Charalambos Kalophonos, Eleni Kourea, Vasiliki Tzelepi, Vassiliki Zolota, Fotios Sampsonas

**Affiliations:** 1 Department of Pathology, Medical School, University of Patras, 26504 Rion, Greece; bosgana.p@gmail.com (P.B.); ekourea@upatras.gr (E.K.); vtzelepi@yahoo.com (V.T.); zol@med.upatras.gr (V.Z.); 2 Department of Anatomy, Embryology and Histology, Medical School, University of Patras, 26504 Rion, Greece; so.nikou@hotmail.com (S.N.); vibra@upatras.gr (V.B.); 3 Division of Oncology, Department of Medicine, Medical School, University of Patras, 26504 Rion, Greece; fotdimitrak@yahoo.gr (F.-I.D.); kalofonos@upatras.gr (C.K.); 4 Department of Pulmonology, Medical School, University of Patras, 26504 Rion, Greece

**Keywords:** adenocarcinoma, LIN28, lung, OCT4, pluripotency

## Abstract

*Background and Objectives:* Lung adenocarcinoma is a leading cause of cancer-related mortality despite recent therapeutic advances. Cancer stem cells have gained increasing attention due to their ability to induce cancer cell proliferation through self-renewal and differentiation into multiple cell lineages. OCT4 and LIN28 (and their homologs A and B) have been identified as key regulators of pluripotency in mammalian embryonic (ES) and induced stem (IS) cells, and they are the crucial regulators of cancer progression. However, their exact role in lung adenocarcinoma has not yet been clarified. *Materials and Methods:* The aim of this study was to explore the role of the pluripotency factors OCT4 and LIN28 in a cohort of surgically resected human lung adenocarcinomas to reveal possible biomarkers for lung adenocarcinoma prognosis and potential therapeutic targets. The expressions of OCT4, LIN28A and LIN28B were analyzed in formalin-fixed, paraffin-embedded tissue samples from 96 patients with lung adenocarcinoma by immunohistochemistry. The results were analyzed with clinicopathologic parameters and were related to the prognosis of patients. *Results:* Higher OCT4 expression was related to an improved 5-year overall survival (OS) rate (*p* < 0.001). Nuclear LIN28B expression was lower in stage I and II tumors (*p* < 0.05) compared to advanced stage tumors. LIN28B cytoplasmic expression was associated with 5-year OS rates not only in univariate (*p* < 0.005), but also in multivariate analysis (where age, gender, histopathological subtype and stage were used as cofactors, *p* < 0.01 HR = 2.592). Patients with lower LIN28B expression showed improved 5-year OS rates compared to patients with increased LIN28B expression. *Conclusions:* Our findings indicate that OCT4 and LIN28B are implicated in lung adenocarcinoma progression and prognosis outcome; thus, they serve as promising prognostic biomarkers and putative therapeutic targets in lung adenocarcinomas.

## 1. Introduction

Adenocarcinoma of the lung (LADC) remains a major cause of cancer-related mortality worldwide despite recent therapeutic advantages. In the 2015 World Health Organization classification, invasive lung adenocarcinoma was further classified into different subtypes, with different prognoses [1] such as EGFR and KRAS mutations, as well as Anaplastic lymphoma kinase (ALK) and ROS1 translocations, which are the most common molecular alterations detected in lung adenocarcinomas and form the basis for targeted therapies [2,3].

Stem cells are either partially differentiated or they partially differentiated cells that can further differentiate into various other cell types and divide indefinitely to produce more of the same stem cell [3]. These cells have unique properties of self-renewal and pluripotency [3]. Stem cells continue to self-renew due to an autoregulated network of transcription factors, which inhibits differentiation and promotes proliferation [4]. Dysregulation of these mechanisms can lead to premature differentiation and/or continuous self-renewal/proliferation of stem cells, which is a well-known hallmark of cancer progression [5].

The LIN28 gene encodes an RNA-binding protein that governs the post-transcriptional regulation of gene expression; thus, it has a crucial role in tissue development [6,7,8,9]. LIN28 controls stem cell self-renewal, thus influencing many cellular functions including cell growth, stem cell differentiation, metabolism and carcinogenesis. The LIN28 family of RNA-binding proteins consists of two highly conserved homologs LIN28A and LIN28B with similar functions [10]. LIN28 binds to let-7 pre-microRNA, and it blocks the biogenesis of mature let-7 in mouse embryonic stem cells (ES) [8]. Various studies link microRNA to critical oncogenic pathways such as the RAS pathways, Myc and JAK-STAT3. In particular, reduced let-7microRNA expression has been observed in many types of cancer, resulting in cancer progression and adverse patient prognosis [9].

OCT4 (also known as POU5F1) is a protein that is encoded in humans by the POU5F1 gene, which is located on chromosome 6p21 [11]. During embryonic development, the expression of OCT4 affects the differentiation of embryonic stem cells that are maintaining their capacity for self-renewal. Also, OCT4 expression is increased in germ cell and embryonic cell tumors, rendering OCT4 a molecular marker of germ cell tumors [12]. 

OCT4 and LIN28 are transcription factors with a key role in pluripotency maintenance in mammalian ES and induced pluripotent stem cells (iPS), regulating, in this way, cancer progression [13,14]. However, their role in lung adenocarcinoma has not yet been fully clarified. 

The aim of this study was to explore the role of pluripotency factors OCT4 and LIN28 (homolog A and B) in a cohort of surgically resected human lung adenocarcinomas to reveal the possible biomarkers for diagnosis and prognosis and the potential therapeutic targets for lung adenocarcinomas. 

## 2. Materials and Methods

Our study included 96 patients who underwent surgical resection for lung adenocarcinoma at the University Hospital of Patras between 2000 and 2009. All tumors were formalin-fixed, paraffin-embedded (FFPE). The hematoxylin and eosin (H&E)-stained slides of the specimens were reviewed by an expert pathologist (PB) to determine the histological subtype, grade and T- and N-stage of the tumor according to the revised 2015 World Health Organization (WHO) classification of Lung Tumors [15]. A representative block was selected for each patient. Non-neoplastic lung parenchyma adjacent to the tumor was also present in most of the blocks (95%). Medical history and clinical outcomes were retrieved from the patients’ records from the Division of Oncology of the University Hospital of Patras. Overall survival was evaluated after an observation period of 5 years (60 months). This study was approved by the Bioethics & Research Committee of the University Hospital of Patras, Greece (approval code: 509 and approval date: 11 July 2019) in full compliance with the guidelines detailed in the Declaration of Helsinki, which provides the “ethical principles for medical research involving human subjects”.

### 2.1. Immunohistochemical Staining

Serial 3 μm tissue sections were cut, mounted on poly-L lysine-coated slides and subjected to immunohistochemical staining. Briefly, the sections were initially dried for 24 h at 60 °C, deparaffinized in xylene and hydrated in gradient alcohol. The antigen was retrieved in Tris/EDTA buffer (pH 9) with a pressure antigen retrieval procedure for 12 min. Next, endogenous peroxidase was inactivated using a peroxidase-blocking solution (0.3% H_2_O_2_) at room temperature for 10 min. The sections were then incubated with the primary antibodies. Information about the primary antibodies, as well as the positive and negative controls used for antibody validation, are shown in Table 1. Immunohistochemical signaling was detected with Dako EnVision polymer (Dako EnVision Mini Flex, Dako Omnis, Angilent Technology Inc., Santa Clara, CA, USA, GV823). Diaminobenzidine (Dako Omnis, Santa Clara, USA, GV823) was used as a chromogen, and Harris Hematoxylin was used for nuclear counterstaining. 

### 2.2. Evaluation of the Immunohistochemical Staining 

The immunoreactivity was assessed by an expert pathologist (PB), who was blinded to the pathological and clinical characteristics of each case. The intensity and the distribution of positively stained cancer cells were evaluated as described below. The localization (nuclear and cytoplasmic) of the stains was also evaluated. The immunoreactivity was calculated with the following formula: The staining immunoreactivity was scored from 0% to 100% (at 5% as intervals) by calculating the proportion of positive tumor cells (more than 1000 cells were counted). The intensity of stained cells was assessed with a three-tiered scale. The overall score was calculated by multiplying the percentage (%) of positive-stained cells by the intensity of the staining, ranging from 0 to 300. Other components of the tumor microenvironment, such as lymphocytes, macrophages and endothelial cells were also evaluated and scored as positive or negative based on the presence or absence of any staining. Microphotographs were obtained by a Lumenera INFINITY HD digital camera (Teledyne Lumenera Co, OTT, Canada) mounted on an Olympus BX41 microscope (Olympus Europa SE & Co, Hamburg, Germany). 

### 2.3. Statistical Analysis 

#### 2.3.1. Associations of OCT4 and LIN28 with Clinicopathological Parameters and the Correlations between Proteins

Statistical analysis was performed using the Statistical Package for Social Sciences version 25 (IBM Corp. Released 2017. IBM SPSS Statistics for Windows, Version 25.0. Armonk, NY, USA). The expression of the markers was associated with clinicopathological parameters. Categorical variables were evaluated with the Chi-square or Fisher exact tests. For ordinal or continuous variables, Kruskal–Wallis or Mann–Whitney tests were used for comparisons between groups. Correlations between the expressions of the proteins were performed using Spearman’s correlation test. 

#### 2.3.2. Survival Analysis

Survival analysis was assessed with Kaplan–Meier plots, and the differences between groups were evaluated with the exact log-rank test. OS and DFS rates were calculated as the interval between the date of diagnosis and the date of death (or the last follow-up). Multivariable analysis was performed with Cox’s proportional hazard regression model. A *p* value < 0.05 was considered statistically significant.

## 3. Results 

### 3.1. Clinical, Demographic and Histopathological Data

The patients’ characteristics are summarized in Table 2. Ninety-six (96) cases were included in this study. The median age of the patients was 65.5 years (range 39–84). Sixteen patients (16.7%) had undergone pneumonectomy, 68 (70.8%) lobectomy, 9 (9.3%) double lobectomy and 3 (3.1%) wedge excision. Two- and three-year survival outcomes were available in 88 patients, and five-year survival outcomes were available in 81 patients. 

### 3.2. Expression of OCT4 in Lung Adenocarcinoma

**Figure 1:**
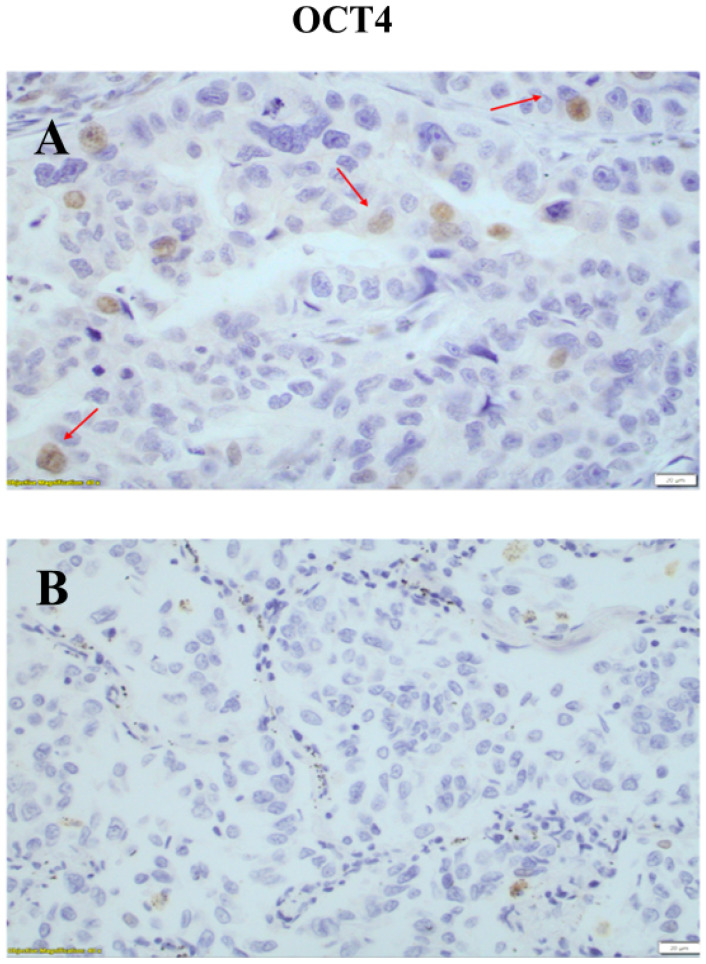
(**A**): Positive nuclear expression of OCT4 in tumor cells (red arrows). Adjacent non neoplasmatic cells were negative for OCT4 expression (magnification ×400 (**B**): Negative nuclear expression of OCT4 in tumor cells. (magnification ×400). Scale bars at 20 μm.

**Figure 2:**
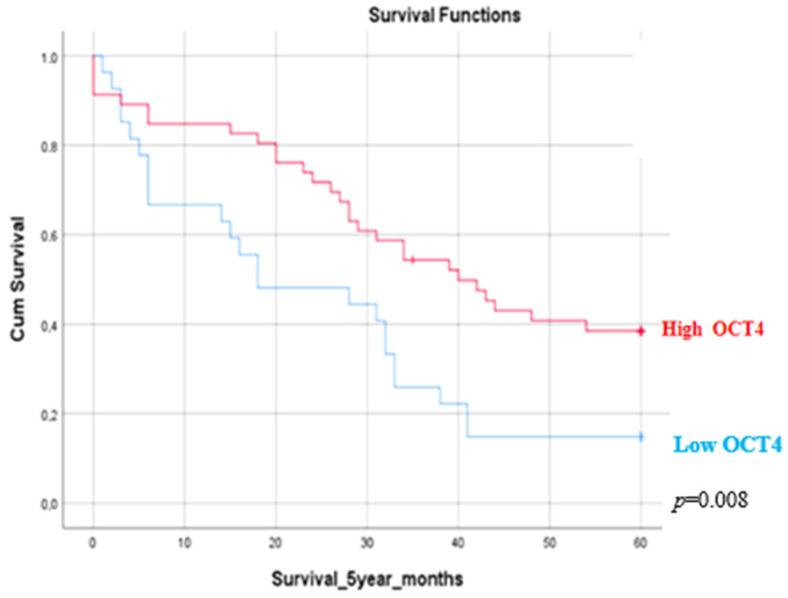
Kaplan–Meier survival estimate. A 5-year survival estimate according to OCT4 expression in patients with lung adenocarcinoma. Patients with higher expression of OCT4 had improved outcomes compared to patients with lower OCT4 expression levels. (*p* = 0.008). A *p*-value of < 0.05 was deemed statistically significant.

Positive OCT4 immunohistochemical staining was observed in the nuclei of the neoplastic cells. The epithelial cells of adjacent non neoplastic lung tissue, lymphocytes and stromal cells were negative for OCT4 (Figure 1). In 61/96 patients, a positive nuclear expression of OCT4 (63.5%) was noted. The immunohistochemical score of OCT4 nuclear expression ranged between 0 and 120 (mean = 4 ± 5) (±SD). The relationships between OCT4 immunohistochemical expression and the clinicopathological data of the patients is presented in Table 3. No significant correlations were observed between OCT4 expression and age (*p* = 0.595), gender (*p* = 0.939), histological subtype (*p* = 0.673) and clinical stage (*p* = 0.542). The immunohistochemical expression of OCT4 in patients with lung adenocarcinoma was associated with 2-, 3-, and 5-year OS rates. A higher nuclear expression of OCT4 was associated with improved 5-year OS rates (*p* = 0.008). Patients with a higher expression of OCT4 had improved outcomes compared to patients with lower OCT4 expression levels (Figure 2). 

### 3.3. Expression of LIN28A in Lung Adenocarcinomas

**Figure 3:**
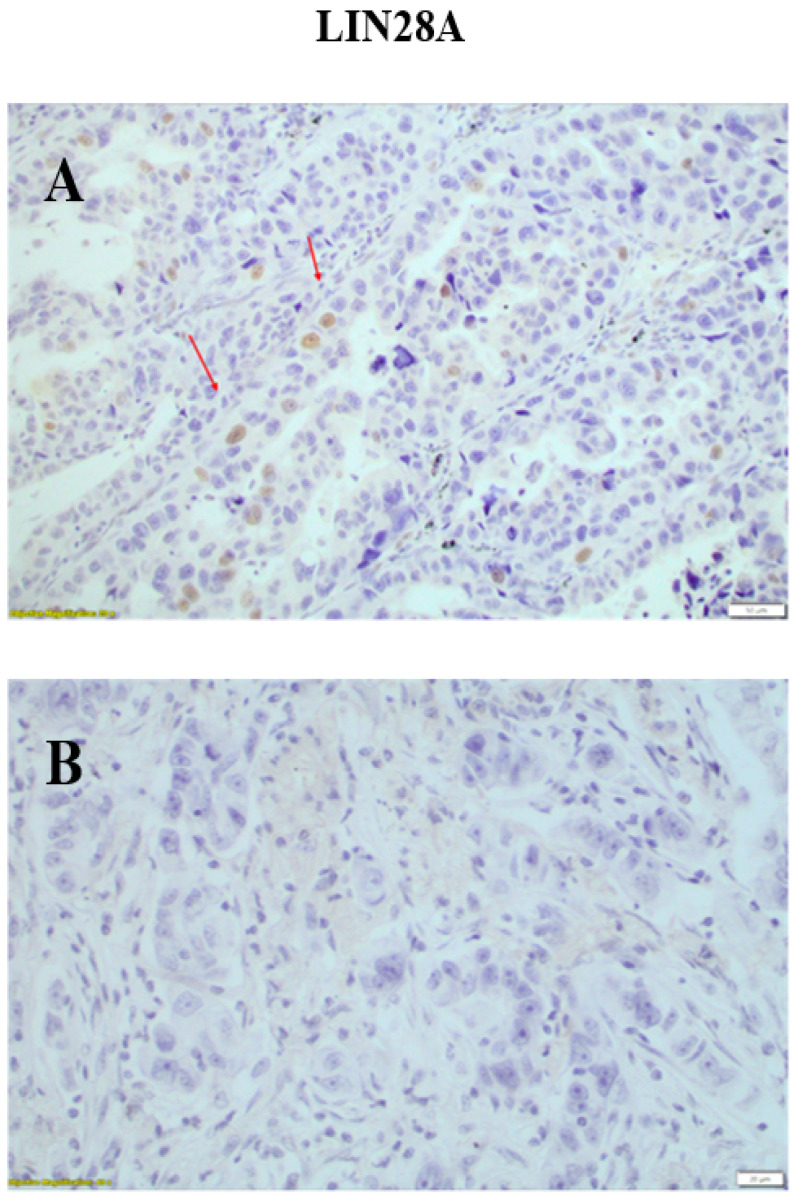
(**A**) Positive nuclear expression of LIN28A in tumor cells (red arrows). The adjacent non neoplasmatic cells were negative (×200). (**B**) Negative LIN28A expression in tumor cells (magnification ×400). Scale bars at 20 μm.

Positive immunohistochemical expressions of LIN28A were observed only in the nuclei of malignant epithelial cells. Epithelial cells of adjacent non neoplastic lung tissue, lymphocytes and stromal cells were negative for LIN28A (Figure 3). In 62/96 patients (64.5%), positive LIN28A nuclear staining was observed, while no LIN28A immunohistochemical expression was observed in 34/96 patients (13.5%) (Table 4).

**Figure 4:**
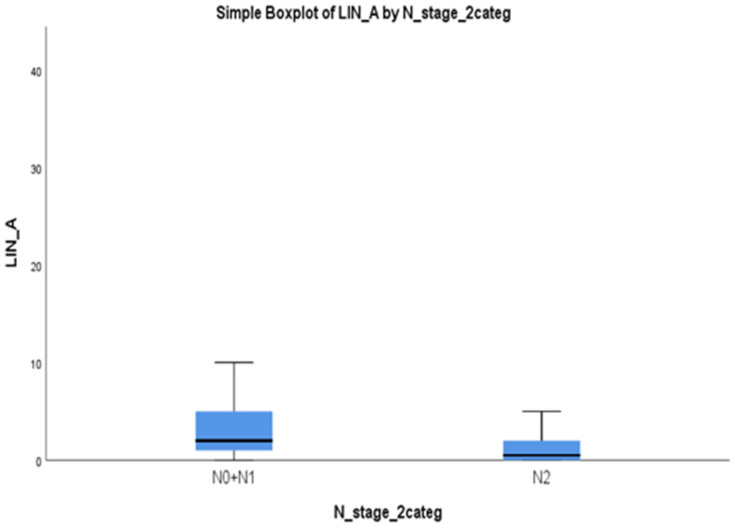
LIN28A expression is associated with lymph node metastasis status. A boxplot analysis indicated that patients with lymph node metastasis (stage N2) have significantly lower LIN28A expression compared to (N0) or (N1) stage (*p* = 0.01). A *p*-value of < 0.05 was statistically significant.

The immunohistochemical score of the positive nuclear immunohistochemical expression ranged between 0 and 75 (median 4 ± 6) (±SD). The relationship between the LIN28A immunohistochemical expression and the clinicopathological data of the patients is presented in Table 3. The immunohistochemical expression of LIN28A was associated with tumor stage and the 5-year survival outcome in patients with lung adenocarcinoma. Patients with metastatic lymph nodes (stage N2) had lower LIN28A expression compared to patients with N0 and N1 disease (*p* = 0.01) (Figure 4). No statistically significant correlations were observed between LIN28A expression and 5-year OS rates (*p* = 0.123), age (*p* = 0.779), gender (*p* = 0.538), histological subtype (*p* = 0.678) and stage (*p* = 0.512). 

### 3.4. Expression of LIN28B in Lung Adenocarcinomas

**Figure 5:**
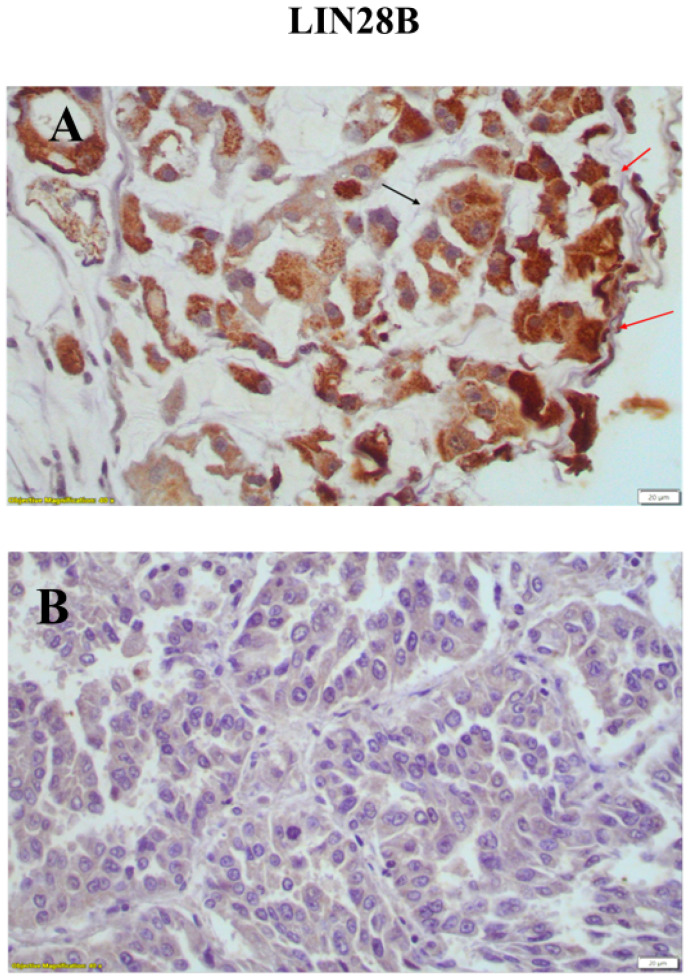
(**A**) Positive cytoplasmic (black arrow) and nuclear (red arrows) expressions of LIN28B in tumor cells (×200). (**B**) Negative nuclear and cytoplasmic expressions in tumor cells (×400). Scale bars at 20 μm.

Positive LIN28B immunohistochemical expression was observed and evaluated in the nucleus and cytoplasm of lung adenocarcinoma cells. In adjacent non neoplastic lung tissue, epithelial cells, lymphocytes and stromal cells were negative for LIN28B (Figure 5). In 68/96 (70.8%) of the patients, positive LIN28B nuclear expression was observed, while no LIN28B nuclear expression was observed in 28/96 (29.2%) patients. In 78/96 patients (81.3%), positive LIN28B cytoplasmic expression was observed, while there was negative LIN28B cytoplasmic expression (Table 5) found in 18/96 patients (18.8%).

**Figure 6:**
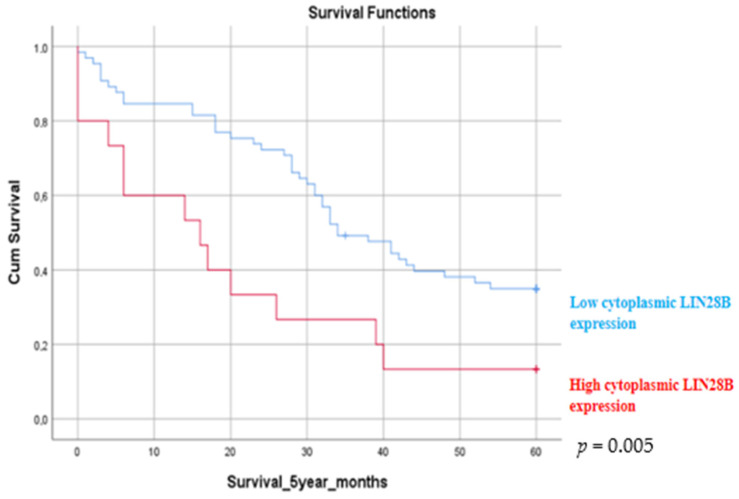
Kaplan–Meier survival estimate. A 5-year survival estimate according to the LIN28B expression in patients with lung adenocarcinoma. Patients with increased cytoplasmic LIN28B expression had poorer 5-year survival rates compared to patients with lower LIN28B expression (*p* = 0.005). A *p*-value of < 0.05 was considered to be statistically significant.

**Figure 7:**
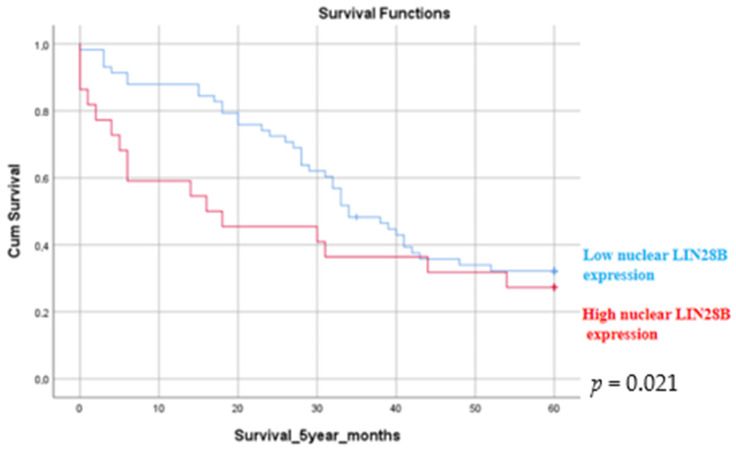
Kaplan–Meier survival estimate. A 2-year survival estimate according to the LIN28B expression in patients with lung adenocarcinoma. Patients with increased nuclear LIN28B expression had poorer 2-year survival rates compared to patients with lower LIN28B expression (*p* = 0.021). A *p* value of < 0.05 was considered to be statistically significant.

The immunohistochemical score of the nuclear LIN28B expression ranged between 0 and 140 (median 14 ± 24) (±SD). The immunohistochemical score of the cytoplasmic LIN28B expression ranged between 0 and 210 (median 68 ± 52) (±SD). The relationships between LIN28B immunohistochemical expression and the clinicopathological data of the patients is presented in Table 5. Nuclear and cytoplasmic LIN28B expression was associated with patient stage and survival. Positive LIN28B cytoplasmic expression was related to 5-year survival in patients with lung adenocarcinoma. Patients with lower LIN28B cytoplasmic expression had a better 5-year survival (*p* = 0.005) rate compared to patients with increased LIN28B expression (Figure 6). No associations between LIN28B cytoplasmic expression and stage (*p* = 0.562), age, gender and histological subtype were observed. Increased LIN28B nuclear expression was statistically significantly associated with poor 2-year survival rates (*p* = 0.021) (Figure 7). The association between LIN28B nuclear expression and stage revealed that patients with early stage lung adenocarcinoma (stages I and II) had statistically significantly lower nuclear expression (*p* = 0.046). No statistically significant association was observed between the nuclear LIN28B expression and age, gender and histological subtype. 

## 4. Discussion

Lung cancer is the leading cause of cancer mortality worldwide [16]. In recent years, significant progress has been made in the discovery of molecular changes; however, the pathogenesis of the disease has not been fully clarified. In this study, we examined the role of pluripotency factor OCT4 and LIN28 (and their A and B homologs) in lung adenocarcinoma in relation to prognosis.

In our study, OCT4 was overexpressed in lung adenocarcinoma, and we showed that a higher OCT4 expression was associated with improved 5-year OS rates. The latest trend in OCT4 research is in connecting OCT4 to epigenetic regulations, which are crucial in cancer development [17,18,19]. However, results about the prognostic role of OCT4 are contradictory. In line with our findings, studies conducted on oral cancer [20] and testicular cancer [21] demonstrated that higher OCT4 expression was associated with better OS rates. It should be noted here that OCT4 has two isomorphs (OCT4A/B). It is possible that the isomorphs that each OCT4 antibody detects target different regions of the OCT4 protein. In contrast, in many types of cancer such as breast cancer and acute myeloid leukemia, increased OCT4 is associated with reduced overall survival rates compared to patients with low OCT4 expression [22,23]. In esophageal carcinoma, increased OCT4 expression was associated with poor prognosis [24]. In lung cancer, a meta-analysis published in 2019 highlighted that increased OCT4 expression was associated with lower overall survival and higher TNM stage [25]. These results contradict our study, where high OCT4 expression in lung adenocarcinoma was associated with better overall survival rates. More studies need to be conducted in large cohorts of patients to elucidate the prognostic role of OCT4 in lung adenocarcinoma.

We also showed that LIN28A is overexpressed in lung adenocarcinoma. However, in our cohort of lung adenocarcinoma patients no statistically significant association was found with aggressive tumor parameters and patients’ prognosis. We found that patients with metastatic lymph nodes (N2) had lower LIN28A expression compared to patients with N0 and N1 disease (*p* = 0.01) which is contradictory with the current literature results. It is possible that the relatively small number of patients in our cohort is a limitation of this analysis. Several studies have revealed that stem cell markers LIN28A and LIN28B regulate gene expression, either by directly binding to messenger RNA (mRNA) or by blocking the biogenesis of Let-7 microRNAs; thus, they are implicated in cancer development [26,27,28,29]. LIN28A, in combination with NANOG, OCT4 and SOX2, can reprogram human somatic cells into pluripotent stem cells. LIN28A also regulates mammalian stem cell self-renewal and promotes tissue repair [30]. LIN28A has been found to be reactivated in ~15% of human cancers and is considered a biomarker of multiple advanced cancers. A high level of LIN28A protein and the subsequent blockage of let-7 biogenesis is associated with tumorigenesis, invasiveness and poor prognosis of malignancies such as lung cancer, liver cancer, breast cancer, gastric cancer and prostate cancer [31]. To the best of our knowledge, the role of LIN28A in human lung adenocarcinoma tissue samples has not been investigated before in the literature. In a recent in vitro study using A549 lung adenocarcinoma cells, LIN28A was linked to MMP2/9 expression. In particular, LIN28A silencing ameliorated MMP2/9 expression levels, as well as metastases. Consequently, LIN28A serves as a marker for tumor development and invasion with potential therapeutic uses [32].

We also observed that LIN28B was overexpressed in lung adenocarcinoma with prognostic value. Increased nuclear and cytoplasmic LIN28B expression was associated with advanced patient stage and reduced survival rates. To the best of our knowledge, there is no other study exploring the role of LIN28A/B in association with prognosis in human lung adenocarcinoma tissue samples. However, in vitro experiments have been conducted in lung cancer cell lines. Our findings agree with the current literature. LIN28B is implicated in the development of multiple tumors such as hepatocellular carcinoma. However, the mechanism of LIN28B activation in cancer remains unclear [33,34]. Overexpression of LIN28A/B has been associated with poor prognosis in many cancers. In a recent meta-analysis including 3772 LIN28A-associated and 1730 LIN28B-associated cases, elevated LIN28A/B expression was significantly associated with poor prognosis in human malignancies [28] such as gastric carcinoma [35], esophageal carcinoma [36], hepatocellular carcinoma [37], breast carcinoma [38], squamous cell carcinoma of the oral cavity [39,40] and adenocarcinoma of the pancreas [41]. A genome-wide analysis study in lung cancer revealed that the H19 gene, which is associated with tumor-cell proliferation, is involved in many types of cancer [42], and it causes an increase in LIN28B expression, which, in turn, promotes lung cancer [43]. In experimental mouse models of non-small cell lung carcinoma, it was found that LIN28B overexpression significantly increased the number of tumor cells, accelerated tumor initiation and resulted in reduced overall survival rates [44]. Also, elevated LIN28B levels have been found in 24% of lung carcinomas harboring the KRAS mutation [44]. Another in vitro study in lung carcinoma cell lines revealed that micro-RNA miR-563 targets and represses LIN28B, thus causing a decrease in cell proliferation [45]. These studies support the prognostic role of LIN28B, as demonstrated in our study, where patients with low nuclear expression had better 5-year survival rates. Our results also highlight LIN28 as an attractive therapeutic target in lung cancer.

## 5. Conclusions

In conclusion, our study shows that the pluripotency factor OCT4 and LIN28 (and their homologs A and B) are implicated in lung adenocarcinoma development and progression with prognostic value. In particular, LIN28B may serve as a marker for dismal patient prognosis in lung adenocarcinoma. Further studies are needed to elucidate their role in lung adenocarcinoma and to explore their potential application as therapeutic agents.

## Figures and Tables

**Table 1:** Information about the antibodies used in this study.

Antibody	Dilution and Incubation Time	Provider	Antigen Retrieval	Positive Control for IHC	Negative Control for IHC
Anti-OCT4 rabbit polyclonal	1:100 Overnight	Abcam Inc. Cambridge, UK	Tris/EDTA buffer (pH 9)	Breast carcinoma	Rabbit immunoglobulin fraction (X0936 DAKO, Hamburg, Germany)
Anti-LIN28A rabbit polyclonal	1:100 Overnight	Abcam Inc. Cambridge, UK	Tris/EDTA buffer (pH 9)	Hepatocellular carcinoma	Rabbit immunoglobulin fraction (X0936 DAKO, Hamburg, Germany)
Anti-LIN28B rabbit polyclonal	1:100 Overnight	Abcam Inc. Cambridge, UK	Tris/EDTA buffer (pH 9)	Hepatocellular carcinoma	Rabbit immunoglobulin fraction (X0936 DAKO, Hamburg, Germany)

**Table 2:** Clinicopathologic characteristics of the patients.

Clinicopathologic Characteristics	N (%)
Total	96 (100)
Male	81 (84.4)
Female	15 (15.6)
Age	<65 years 48 >65 years 48
Histology	
Solid	44 (45.8)
Lepidic	9 (9.4)
Acinar	23 (24)
Papillary	7 (7.3)
Colloid	7 (7.3)
Enteric	5 (5.2)
Fetal	1 (1)
Stage	
I	31 (32.3)
II	26 (27.1)
III	26 (27.1)
IV	8 (8.3)
NA	5 (5.2)
pT stage	
T1	14 (14.6)
T2	55 (57.3)
T3	16 (16.7)
T4	10 (10.4)
NA	1 (1.0)
pN stage	
N0	37 (38.5)
N1	33 (34.4)
N2	20 (20.8)
NA	6 (6.3)

Abbreviations—NA: data not available or unknown; pT: pathologic tumor status; pN: pathologic node status; Stage: as specified by the AJCC 8th edition; and AJCC: American Joint Committee on Cancer.

**Table 3:** Relationship between the immunohistochemical expression of OCT4 and clinicopathological data.

OCT4 Nuclear				
		61 (63.5%)	4 ± 5	
Age	<65	48	4 ± 4	0.405
	>65	48	5 ± 7	
Gender	Male	81	4 ± 6	0.879
	Female	15	4 ± 4	
Histological subtype	Solid	44	4 ± 4	0.911
	Non-solid	52	5 ± 7	
pT	pT1	14	4 ± 4	0.435
	pT2	55	5 ± 6	
	pT3	16	4 ± 4	
	pT4	10	3 ± 3	
pN	pN0	37	5 ± 4	0.483
	pN1	33	4 ± 7	
	pN2	20	3 ± 3	
Stage	1	31	4 ± 4	0.412
	2	26	6 ± 9	
	3	26	3 ± 3	
	4	8	5 ± 6	

Abbreviations—pT: pathologic tumor status; pN: pathologic node status; Stage: according to the AJCC 8th edition; and AJCC: American Joint Committee on Cancer.

**Table 4:** Relationships between the immunohistochemical expression of LIN28A and clinicopathological data.

LIN28A				
		62 (64.5%)	4 ± 6	
Age	<65	48	4 ± 8	0.567
	>65	48	3 ± 3	
Gender	Male	81	3 ± 6	0.201
	Female	15	5 ± 7	
Histological subtype	Solid	44	3 ± 3	0.987
	Non-solid	52	4 ± 7	
pT	pT1	14	4 ± 4	0.468
	pT2	55	4 ± 6	
	pT3	16	4 ± 7	
	PT4	10	3 ± 4	
pN	pN0	37	5 ± 8	0.098
	pN1	33	4 ± 5	
	pN2	20	1 ± 2	
Stage	1	31	5 ± 8	0.396
	2	26	3 ± 2	
	3	26	3 ± 6	
	4	8	2 ± 2	

Abbreviations—pT: pathologic tumor status, pN: pathologic node status, Stage: according to the AJCC 8th edition; and AJCC: American Joint Committee on Cancer. A *p* value of < 0.05 was considered to be statistically significant. Significant *p*-values appear in bold.

**Table 5:** Relationships between the nuclear and cytoplasmic LIN28B immunohistochemical expression and clinicopathological data.

		LIN28B Nucleus			LIN28B Cytoplasm		
Total = 96		Positive (%)	Mean ± SD	*p*-Value	Positive (%)	Mean ± SD	*p*-Value
		68 (70.8%)	14 ± 24		78 (81.3%)	68 ± 52	
Age	<65	48	14 ± 23	0.357	48	61 ± 50	0.423
	>65	48	15 ± 25		48	76 ± 53	
Gender	Male	81	15 ± 26	0.87	81	68 ± 51	0.759
	Female	15	12 ± 11		15	70 ± 55	
Histological subtype	Solid	44	15 ± 25	0.866	44	61 ± 50	**0.034**
	Non-solid	52	14 ± 23		52	75 ± 53	
pT	pT1	14	30 ± 49	0.454	14	96 ± 57	0.879
	pT2	55	12 ± 14		55	59 ± 45	
	pT3	16	14 ± 21		16	75 ± 63	
	pT4	10	7 ± 10		10	72 ± 52	
pN	pN0	37	16 ± 26	0.139	37	70 ± 57	0.059
	pN1	33	14 ± 19		33	56 ± 42	
	pN2	20	7 ± 10		20	79 ± 54	
Stage	1	31	14 ± 25	0.095	31	72 ± 54	0.489
	2	26	17 ± 20		26	55 ± 46	
	3	26	6 ± 9		26	67 ± 52	
	4	8	20 ± 27		8	84	

Abbreviations—pT: pathologic tumor status; pN: pathologic node status; Stage: according to the AJCC 8th edition; SD: standard deviation (SD); and AJCC: American Joint Committee on Cancer. A *p* value of < 0.05 was considered to be statistically significant. Significant *p*-values appear in bold.

## Data Availability

The data presented in this study are available on request from the corresponding author. The data are not publicly available due to privacy.

## Abbreviations

Octamer binding transcription factor 4 (OCT4); RNA-binding protein LIN28 (LIN28); cancer stem cells (CSCs); embryonic stem cells (ES); induced pluripotent stem cells (iPS); overall survival (OS); hazard ratio (HR); lung adenocarcinoma (LADC); formalin-fixed, paraffin-embedded (FFPE); data not available or unknown (NA); standard deviation (SD), epidermal growth factor receptor (EGFR); Kirsten rat sarcoma virus (KRAS); and repressor of Silencing 1 (ROS1).
